# A strategy to characterize chlorophyll protein interaction in LIL3

**DOI:** 10.1186/s13007-018-0385-5

**Published:** 2019-01-05

**Authors:** Astrid Elisabeth Mork-Jansson, Lutz Andreas Eichacker

**Affiliations:** Centre for Organelle Research, Faculty of Science and Technology, University of Stavanger, 4021 Stavanger, Norway

**Keywords:** LIL3, Chl *a*, Reconstitution, DTT, DDM, DLS, NanoDSF, MST

## Abstract

**Background:**

The function of proteins is at large determined by cofactors selectively bound to protein structure. Without chlorophyll specifically bound to protein, light harvesting and photosynthesis would not be possible. The binding of chlorophyll to light harvesting proteins has been extensively studied in reconstitution assays using proteins expressed in vitro; however, the mechanism of the reconstitution reaction remained unclear. We have shown that membrane integral light-harvesting-like protein, LIL3, binds chlorophyll *a* with a Kd of 146 nM in vitro by thermophoresis. Here, reconstitution of chlorophyll binding to LIL3 has been characterized by four different methods.

**Results:**

Structural changes in the reconstitution process have been investigated by light-scattering and differential Trp-fluorescence. For characterization of the chlorophyll binding site at LIL3, the analysis of LIL3 mutants has been conducted using native PAGE and thermophoresis. We find that the oxidized state of dithiothreitol is the essential component for reconstitution of chlorophyll binding to LIL3 in *n*-Dodecyl β-d-maltoside micelles at RT. Chlorophyll increased the polydispersity of the micellar states while dithiothreitol maintained LIL3 in a partially unfolded state at RT. Dimerization of LIL3 was abolished if amino acids N174, R176, and E171 were mutated to Ala; while, chlorophyll binding to LIL3 was abolished in mutant N174A, but retained in E171A, and R176A albeit at an about six- and five-fold decreased dissociation constant. Results show that N174 of LIL3 is essential for binding chlorophyll *a*.

**Conclusions:**

Chlorophyll binding to LIL3 can be shown by thermophoresis, and native gel electrophoresis, while analysis of reconstitution conditions by dynamic light scattering and differential scanning fluorometry are of critical importance for method optimization.

**Electronic supplementary material:**

The online version of this article (10.1186/s13007-018-0385-5) contains supplementary material, which is available to authorized users.

## Background

The family of light-harvesting-like proteins (LIL) are regarded as evolutionary descendant of cyanobacterial high-light inducible protein (HLIP) [1]. HLIPs were postulated to have undergone endosymbiotic gene transfer and modification, resulting in the nuclear encoded one-helix proteins (OHP) and two-helix stress-enhanced protein (SEP) [1]. Genetic analysis specifies LIL3 as a SEP and as a precursor of the four helix non-photochemical quenching protein (PSBS) and of the four helix light-harvesting complex protein (LHC). It has been shown that LIL3 accumulation precedes chlorophyll (Chl) synthesis and assembly in protein complexes is essential for synthesis of Chl and tocopherol; although, the function of LIL3 has not been resolved [2, 3].

Reconstitution is a method extensively employed in the study of pigment binding to protein over the last 30 years. In 1987, delipidated LHC2 was isolated from thylakoid membranes and was reconstituted with pigments and xanthophyll’s [4]. Three years later, reconstitution of recombinant LHC2 was shown to be dependent on chlorophyll *a* (Chl *a*) and carotenoid binding [5]. Several groups reported thereafter about selective pigment binding sites using recombinant LHC proteins [5–8]. Chl *a* binding in the absence of carotenoids was shown for recombinant LIL3 [9, 10], and also for the recombinant reaction center protein CP43 [11]. For Chl binding to members of the LHCs, there is broad consensus that binding is mediated by concerted action of a number of amino acids termed the LHC motif [12, 13]. In one report, the LHC motif in LIL3 was postulated to structurally anchor Geranylgeraniol-reductase (GGR) to the membrane, and to be responsible for the oligomerization of GGR [14].

The conserved LHC motif is an overall hydrophobic amino acid sequence composed of 22 amino acids with two charged amino acids: glutamic acid (E), arginine (R) and three glycine (G) residues within the sequence ELINGRLAMLGFLGFLVPELIT [13] and a consensus motif E–X–X–H/N–X–R or R–X–N/H–X–X–E found at Chl binding sites [12, 13, 15, 16]. In LHC, residues E and H/N were described to be responsible for coordination of the central Mg^2+^ ion of Chl. The anionic carbonyl group in E and the guanidinium group in R residues are discussed to play an important role for salt ion paring in E139–R142, E65–R185 and E180–R70 [17]. We applied dynamic light scattering and nano-DSF for component analysis during in vitro reconstitution and find that oxidized DTT is the essential component in reconstitution of Chl *a* binding to LIL3.2 (LIL3). We show that oxidized DTT maintains LIL3 in a partially unfolded state in the presence of *n*-Dodecyl β-d-maltoside (DDM) micelles at RT. Using microscale thermophoresis (MST) and native PAGE, amino acid N174 is shown to be essential for Chl *a* binding to LIL3, while amino acids E171 and R176 show residual activity for reconstitution of Chl *a* binding to the LIL3 mutants.

## Results

### DDM and DTT are key components to establish reconstitution of LIL3

**Fig. 1:**
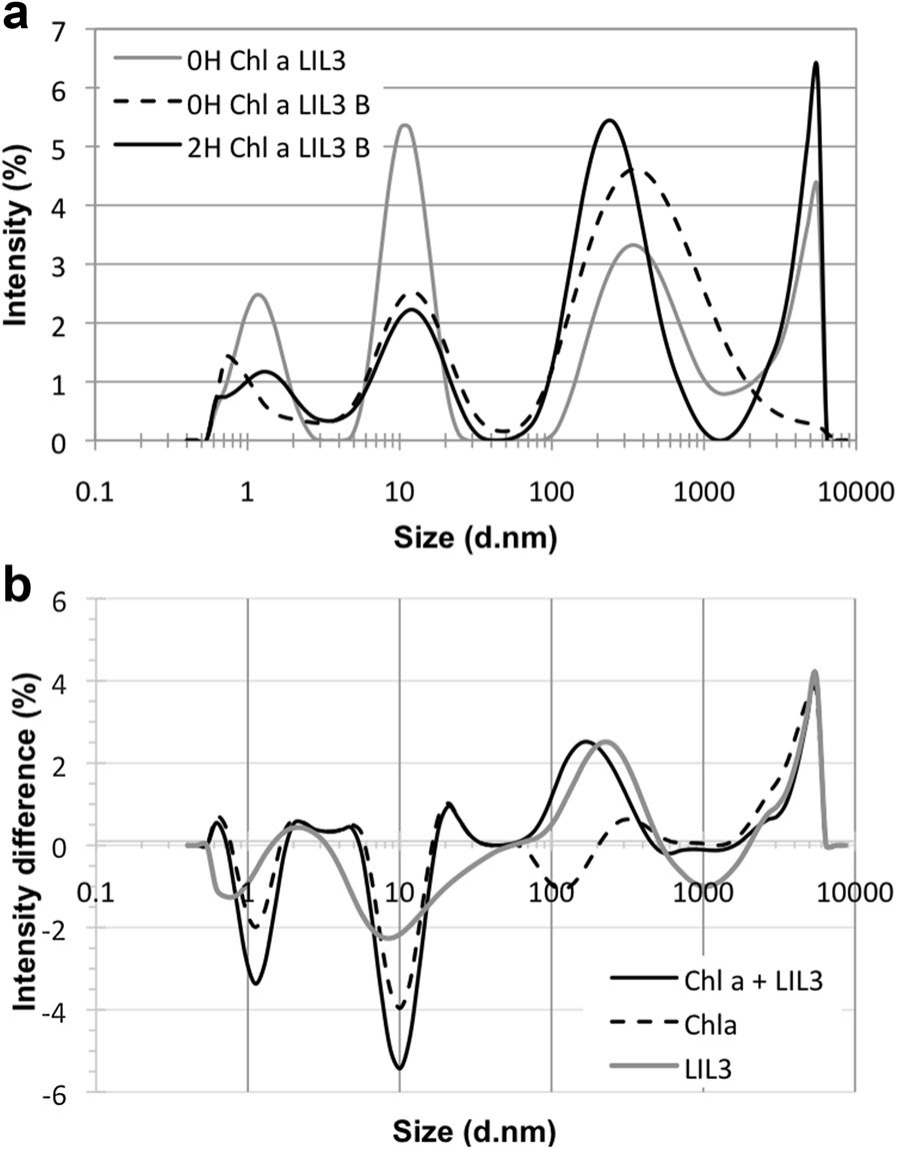
LIL3 is co-localized with DDM micelles when reconstituted with Chl *a*. LIL3.2 was reconstituted with Chl *a* in the presence of 6 mM DDM micelles and 100 mM DTT and the intensity distribution profiles [diameter (d. nm)] of reconstitution steps were recorded upon assembly of the reaction components (0H), 1 min after boiling (0H B), and 2 h after boiling (2H B) (**a**). Difference spectra represent the intensity distribution difference 2 h after boiling, between reconstitution assays containing reaction components DTT and DDM plus Chl *a* and LIL3 minus reaction assays containing DTT/DDM (Chl *a* + LIL3, black line), or DTT/DDM, and LIL3 (Chl *a*, broken black line) or DTT/DDM, and Chl *a* (LIL3, grey line) (**b**)

The reconstitution of the membrane integral light-harvesting-like protein LIL3, was investigated in reconstitution buffer at pH 11. The size of reconstitution buffer components DDM, DTT, Chl *a*, and LIL3 and their interactions were investigated. Investigations were conducted after the 2 h reconstitution incubation as well as before, and after heating of the reaction components (Fig. 1). Samples typically showed a multimodal polydisperse distribution profile with three main peaks. When both Chl *a* and LIL3 were present during reconstitution, the intensity of DTT/reconstitution components at around 1 nm and DDM micelles at around 10 nm decreased, while particles in the distribution range between 100 and 1000 nm increased with a peak at 283.6 ± 48 nm (Fig. 1a, b, Table 1 and controls Additional file 2: Fig. S1, Additional file 3: Fig. S2, Additional file 4: Fig. S3, Additional file 5: Fig. S4). In addition, differential analysis shows that in the presence of Chl *a*, LIL3 and both components particles in the range of 2–3 nm increased (Fig. 1b). Data indicated that during reconstitution at RT, Chl *a* is dissolved by DDM, while LIL3 is dissolved by DTT and in part by DDM upon heating. During the 2 h incubation at RT, Chl *a*/DDM is increased in the distribution range of the LIL3/DTT fraction, indicating that particles fuse (Fig. 1b). Control reactions enabled a differentiation between the multimodal polydisperse distribution profiles. For DDM in reconstitution buffer, a mean hydrodynamic diameter (h_d_) of about 1.2 ± 0.03 nm, 10.7 ± 0.82, and 470.07 ± 189.5 nm was recorded in the absence and presence of DTT (Additional file 2: Fig. S1, Additional file 4: S3, Additional file 1: Table S1). Intensity and distribution range of components in the size range of about 1 and 10 nm were stable, while larger assembly of components shifted to a h_d_ of 361.33 ± 39.342 nm upon heating and 304.4 ± 35.9 nm upon incubation for 2 h at RT (Additional file 2: Fig. S1, Additional file 1: Table S1). In the absence of DDM but presence of DTT, particles with a distribution range of about 10 nm diameter were missing, but components in the 1 nm range showed an about threefold increase throughout all tested conditions, and components with a h_d_ of about 377.4 ± 31.6 nm diameter at time-point zero, shifted to 558.1 ± 60 nm upon heating and 2 h incubation (Additional file 2: Fig. S1, Additional file 1: Table S1). This showed that DDM formed micelles with a h_d_ of 9.61 ± 1.236 nm upon completion of the reconstitution protocol in reconstitution buffer, whereas peaks in the range of about 1 nm were dominated by DTT and reconstitution buffer components (Fig. 1 and Additional file 2: Fig. S1, Additional file 3: Fig. S2, Additional file 4: Fig. S3, Additional file 5: Fig. S4). Peaks in the range of 100 to 1000 nm were found to be variable in diameter and sensitive to either DDM and DTT (Additional file 2: Fig. S1 and Additional file 4: S3). In the presence of Chl *a* and LIL3, both DTT and DDM, showed a differentiated change in particle distribution of reconstitution assay components. Chl *a* did hardly influence the final distribution of particles in the reconstitution assay in the presence of DTT alone; but, stabilized the formation of the DDM micelles at about 11.6 ± 0.13 nm. However, in the presence of both DTT and DDM, addition of Chl *a* resulted in a generally decreased intensity of the particles´ distribution profiles upon heating and the DDM specific signal partly recovered during the 2 h incubation indicating that Chl *a* interacted with DDM micelles (Additional file 3: Fig. S2). In contrast, LIL3 decreased distribution profiles in the 1 nm and increased it in the 100–1000 nm range in the presence of DTT only; although, hardly any changes where determined in the presence of DDM only, indicating that LIL3 interacted with DTT (Additional file 3: Fig. S2). The influence of DTT was investigated by thermal unfolding of LIL3 using nanoDSF-analysis.

**Table 1:** Mulitmodal polydisperse mean peak intensity profiles

Sample	DDM DTT			DDM DTT LIL3			DDM DTT Chl *a*			DDM DTT Chl *a* LIL3		
Peak	1	2	3	1	2	3	1	2	3	1	2	3
0H	10.33 ± 0.14	359.56 ± 18.03	1.10 ± 0.02	10.21 ± 0.10	441.13 ± 96.89	1.11 ± 0.03	11.34 ± 0.22	332.17 ± 40.82	1.22 ± 0.04	11.42 ± 0.50	399.23 ± 87.55	
0H B	10.46 ± 0.18	455.47 ± 118.62	1.11 ± 0.01	366.53 ± 128.27	10.53 ± 0.32	1.13 ± 0.02	13.85 ± 3.76	443.20 ± 318.05		559.30 ± 142.94	12.64 ± 0.94	1.15 ± 0.58
2H B	10.58 ± 0.08	358.27 ± 10.63	1.18 ± 0.03	262.23 ± 40.05	10.68 ± 0.14	1.18 ± 0.08	12.24 ± 1.20	567.3 ± 359.54	0.99 ± 0.03	283.6 ± 47.97	4293.3 ± 622.73	12.38 ± 2.00

The diameter of particles (values in nm) was recorded from samples in reconstitution buffer (methods) in the presence of DDM and DTT (DDM DTT), and supplemented with either LIL3 (DDM, DTT, LIL3), Chl *a* (DDM, DTT, Chl *a*), or both (DDM, DTT, Chl *a*, LIL3). Sample diameters were recorded after assembly of the reaction components (0H), 1 min after heating (100 °C) (0H B), and 2 h after heating (2H B). The diameters from the mulitmodal polydisperse mean peak intensity profiles of each sample were arranged according to the intensity values as 1 (highest), 2 (middle), 3 (lowest) intensity (peak)

### LIL3 shows a stepwise thermal unfolding in the presence of oxidized DTT

**Fig. 2:**
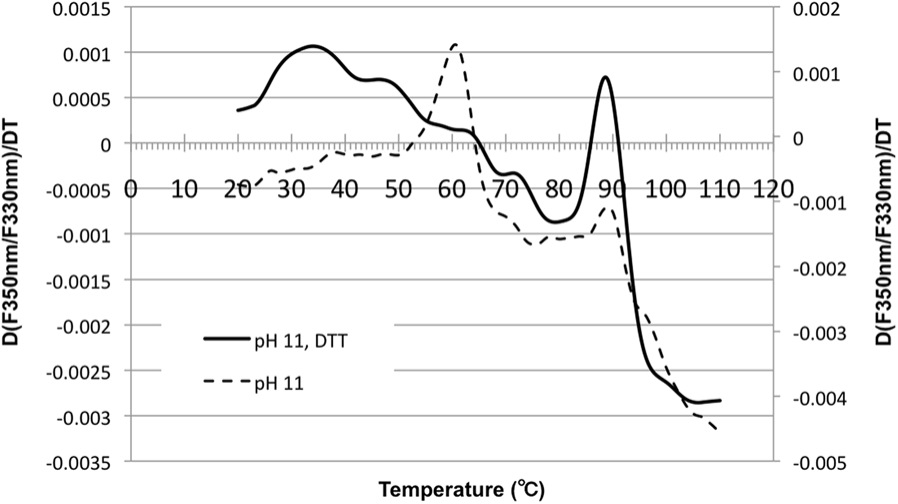
Oxidized DTT induces a stepwise thermal unfolding of LIL3. The first derivative was calculated from the fluorescence ratio changes [Δ(*F*350 nm/*F*330 nm)/Δ*T*] determined as a function of temperature (°C), for LIL3 solubilized by DDM in reconstitution buffer in the absence (pH 11) and presence of DTT at pH 11 (pH 11, DTT)

**Fig. 3:**
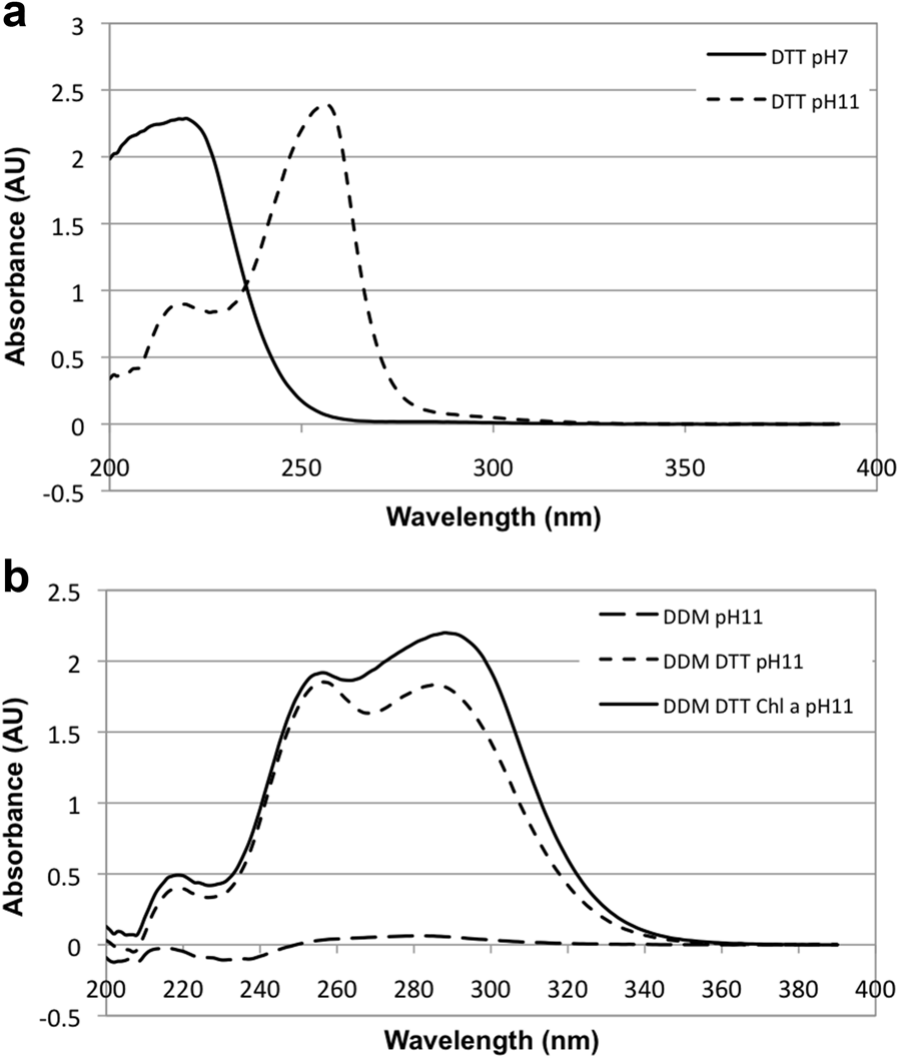
DTT is oxidized at pH 11 and maintains solubility of LIL3. Absorbance spectra were recorded from 200 to 400 nm in a Shimadzu UV–VIS 2401PC spectrophotometer. DTT was dissolved in dH_2_O (DTT, pH 6, solid line) and reconstitution buffer (DTT, pH 11, broken line) (**a**). Spectra of LIL3 were recorded upon solubilization in DDM in reconstitution buffer at pH 11 in the absence (DDM) and presence of DTT (DDM, DTT) and upon reconstitution with Chl *a* (DDM, DTT, Chl *a*) (**b**)

Thermal unfolding of LIL3 was investigated under reconstitution conditions in the presence and absence of DTT upon solubilization in reconstitution buffer (Fig. 2, pH 11). Unfolding of LIL3 was recorded via the temperature dependent changes in Trp-fluorescence, at 350 nm and 330 nm. The ratio between both emissions was determined and the first derivative of the graph was plotted and analyzed. Typically, two transition temperatures were found (Fig. 2). A fluorescence maximum was determined at 60 °C, indicating an exposure of Trp-residues to be energetically coupled to unfolding of a LIL3 domain (Fig. 2, pH 11). A second maximum at 90 °C indicated complete denaturation of the LIL3 protein. In the presence of DTT, the second temperature transition of LIL3 was maintained at 90 °C and the transition was more pronounced indicating a more narrow temperature range and exposure of a higher number of Trp residues. However, the thermal transition at 60 °C was no longer detectable, and unfolding was already initiated at around 30 °C, with transitions extending over a broader temperature range. Here, four to five phases of thermal unfolding were recorded with maxima at 36, 48, (58), 62, and 72 °C (Fig. 2, pH11, DTT). In contrast, two thermal transitions at 55/63 °C and 88/90 °C were determined for LIL3 in the presence and absence of DTT, when unfolding was investigated at pH 6.0 (Additional file 6: Fig. S5). This indicated that DTT selectively affected the unfolding of LIL3. Since DTT should be deprotonated at pH 11, we spectroscopically controlled the reduction state of DTT at pH 6.0 and 11 (Fig. 3a). Spectra showed that DTT was deprotonated and present in its oxidized state at pH 11 (Fig. 3a). The oxidation state of DTT was also not affected by the presence of LIL3 in the absence or presence of Chl (Fig. 3b). It was therefore concluded that oxidized DTT results in a stepwise unfolding of LIL3 in the range of 33.6–88.4 °C (Fig. 2). In contrast, exposure of Trp residues was found within a very narrow thermal transition in the presence of reduced DTT or its absence at pH 6.0, relative the broad and thermally dispersed transition steps in the presence of DTT at pH 11. This suggested that oxidized DTT affects LIL3 by chemically lowering the energetic requirement for unfolding. With respect to the folding mechanism of LIL3 during reconstitution and hence after heating in the reconstitution buffer, data suggested that oxidized DTT enables LIL3 to fold more slowly to establish a stable interaction with Chl *a* during the 2 h reconstitution reaction at RT. Finally, spectroscopic analysis showed hardly any contribution of the LIL3 protein when it was exposed to reconstitution conditions in the absence of oxidized DTT (Fig. 3, LIL3, b). This unexpected finding indicated that oxidized DTT maintained the solubility of LIL3 in a dissolved and non-aggregated state despite the presence of DDM during the heating step of the reconstitution reaction. It was therefore investigated whether the stability of DDM micelles is altered by the components of the reconstitution reaction.

### The stability of LIL3 in DDM micelles increases in the presence of Chl *a*

**Fig. 4:**
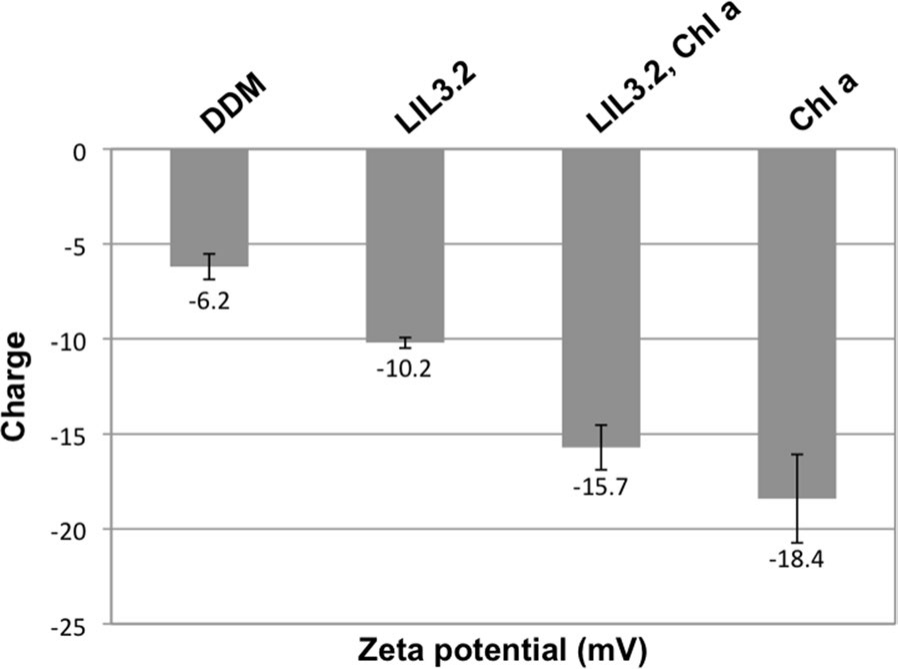
LIL3 in DDM micelles is more stable in the presence of Chl *a*. The charge distribution profile (zeta potential, mV) of DDM was recorded by electrophoretic light scattering (DDM) and compared to LIL3 solubilized in DDM (LIL3), LIL3 reconstituted with Chl *a* (LIL3, Chl *a*), and Chl *a* solubilized in DDM (Chl *a*) under reconstitution conditions

The stability of a suspension of particles against aggregation can be determined by electrophoretic light scattering. The method is based on the particles´ zeta potential causing particle motion in an oscillating electric field. Here, the stability of DDM micelles was determined and compared to micelles containing LIL3, Chl *a*, and LIL3 plus Chl *a* upon completion of reconstitution. None of the DDM micelles showed high oscillating mobility in the electric field indicative for stable dispersions. Generally, the presence of Chl *a* contributed most to the stabilization of the DDM micelles. The relative highest value was determined with − 6.2 mV for DDM micelles, and the value was lowered in the presence of LIL3 with − 10.2 mV (Fig. 4). For LIL3 reconstituted with Chl *a*, a low standard deviation plus low zeta potential of − 15,7 mV was noted relative to a high standard deviation and the lowest zeta potential of − 18,4 mV for Chl *a* in DDM micelles. Data suggest that binding of Chl *a* to LIL3 contributes to a stabilization of DDM micelles against aggregation. To determine the specificity of Chl *a* binding, MST analysis was performed using LIL3 substitution mutants.

### LIL3 mutant N174A does not interact with Chl *a*

**Fig. 5:**
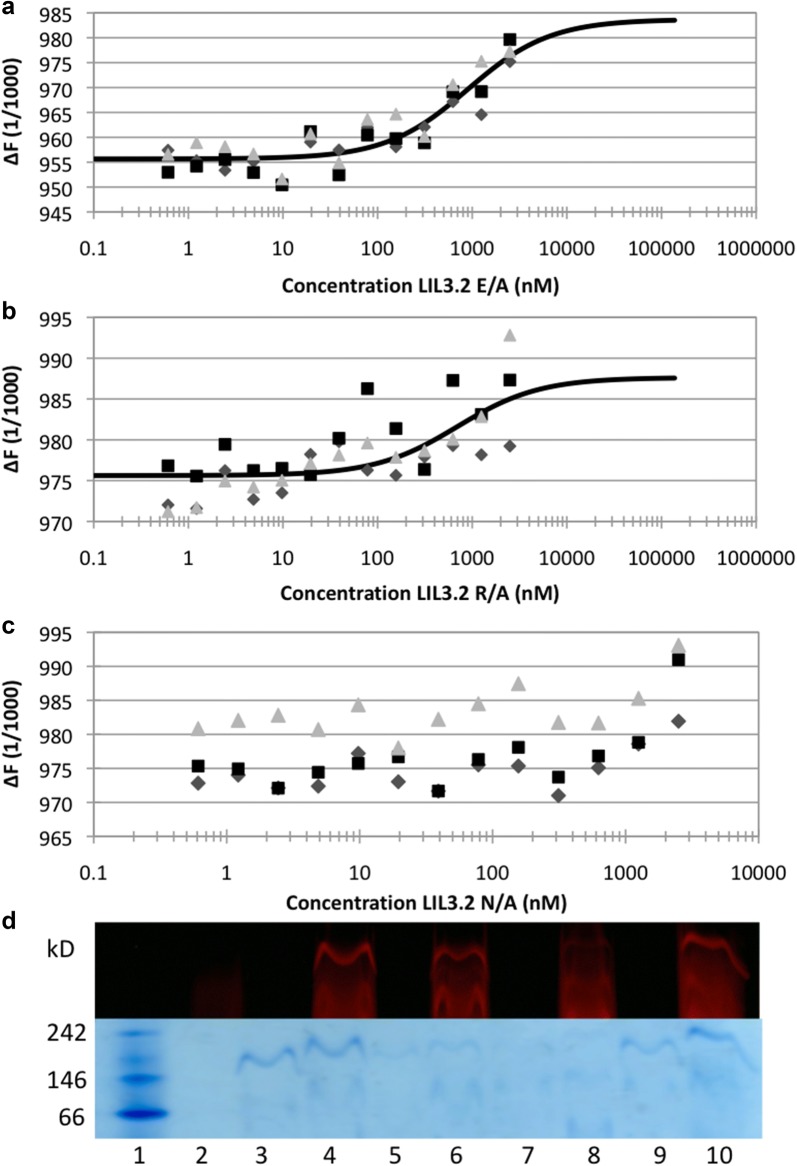
LIL3 mutants resolve Chl *a* binding to N174. Lil3 mutants E171A (**a**), R176A (**b**), and N174A (**c**) were solubilized in DDM micelles (6 mM) at increasing protein concentrations 0.61 nM–2.5 µM in the presence of a constant concentration (120 nM) of Chl *a*. The fluorescence difference from three MST measurements was plotted against the LIL3 mutant concentrations. Recombinant LIL3 WT and mutants were reconstituted with Chl *a* and assay components were isolated using native 3–12% LN-PAGE. The mobility of Chl *a* in native gels was determined by laser excitation/emission scanning at 680/700 nm (**d**). The mobility of LIL3 WT and mutants in the gels were determined by in-gel staining using colloidal Coomassie (**d**). Lane numbers refer to native mark (1), and reconstitution assays containing LIL3 (30 µM), Chl *a* (6 µM). Lanes: Native Mark (1), Chl *a* (2), LIL3.2 WT (3), LIL3.2 WT reconstituted (4), LIL3.2 E171A (5), LIL3.2 E171A reconstituted (6), LIL3.2 N174A (7), LIL3.2 N174A reconstituted (8), LIL3.2 R176A (9), LIL3.2 R176A reconstituted (10)

The interaction of Chl *a* with LIL3 during reconstitution was investigated in LIL3 mutants. Amino-acids E171, N174 and R176 which are conserved in LIL3 and which are part of the LHC motif were exchanged against alanine. The strength for interaction between Chl *a* and LIL3 was investigated in microscale thermophoresis (MST) experiments by titration of a constant Chl *a* concentration with increasing concentrations of mutant LIL3 protein (0.15 nM–2.54 µM) (methods). Here, Chl *a* served as a fluorescent reporter to monitor the molecular changes in size, charge or hydration shell of DDM micelles upon interaction with LIL3. After the 2-h dark incubation of the reconstitution assays MST experiments revealed an average Kd value of 697 nM, and 901 nM for binding of Chl *a* to LIL3 mutants R176A and E171A, respectively (Fig. 5a, b). In contrast, no binding of Chl *a* to LIL3 mutant N174A could be determined (Fig. 5c). Kd values for mutants E171A (Fig. 5a) and R176A (Fig. 5b) were high with an about six-, and five-fold lower binding strength for Chl *a* with the mutant LIL3 relative to WT (146 nM). In order to verify this finding by an independent method, the components of the reconstitution reactions were applied to native PAGE (Fig. 5d). Data showed that the binding of Chl *a* to LIL3 was only maintained in a protein complex of about 240 kD when amino acids N174 and R176 were available in LIL3 mutant E171A (Fig. 5d6) or amino acids N174 and E171 in LIL3 mutant R176A (Fig. 5d10); while, amino acids E171 and R176 were not sufficient for Chl *a* binding in the LIL3 mutant N174A (Fig. 5d8). It was therefore concluded that amino acid N174 is essential to establish an interaction of Chl *a* with LIL3 protein. Finally, the interaction of all LIL3 mutant protein with fluorescently labelled WT LIL3 (LIL3.2-NT647) was investigated (Additional file 7: Fig. S6). Interestingly, none of the LIL3 mutants retained an interaction with WT LIL3 indicating that the functional group in either amino acid E171, N174, or R176 contributes to stabilize the LIL3 dimer (Additional file 7: Fig. S6). Data furthermore indicated that the interaction of amino acid N174 with Chl *a* in LIL3 mutants E171A and R176A reflects binding of Chl *a* to a LIL3 monomer.

## Discussion

It has been shown that reconstitution of LIL3 can be well documented using nanoscale thermophoresis and native PAGE. In a reconstitution buffer containing DDM and oxidized DTT, the LIL3 protein remains soluble and in a partially unfolded state upon heat denaturation whereby its interaction with Chl *a* can be studied at RT.

### Helical regions containing Trp in LIL3 interact with transmembrane domains

Complete denaturation of LHC2 had been described already [4], as an essential requirement for reconstitution, and the protocol has been maintained in all LHC proteins reconstituted thereafter [4, 5, 18]. However, the principle of unfolding/refolding of the transmembrane domains in LHC upon heat denaturation and especially the question how this could influence the mechanism of reconstitution is not understood well [19]. The dynamic scanning of protein endogenous Trp-fluorescence changes during continuous heating of a protein sample has been developed in recent years as a superior label-free method to study the dynamics of protein folding (Application note, Prometheus, protein stability, Nanotemper Technologies GmbH). The unfolding reaction of the protein is hereby determined as change in the Trp-fluorescence at 350 nm and 330 nm and the first derivative of the ratio between both wavelengths is analyzed against the temperature variable. Hereby, determination of the ratio of the fluorescence yield has the advantage to cancel out background noise.

**Fig. 6:**
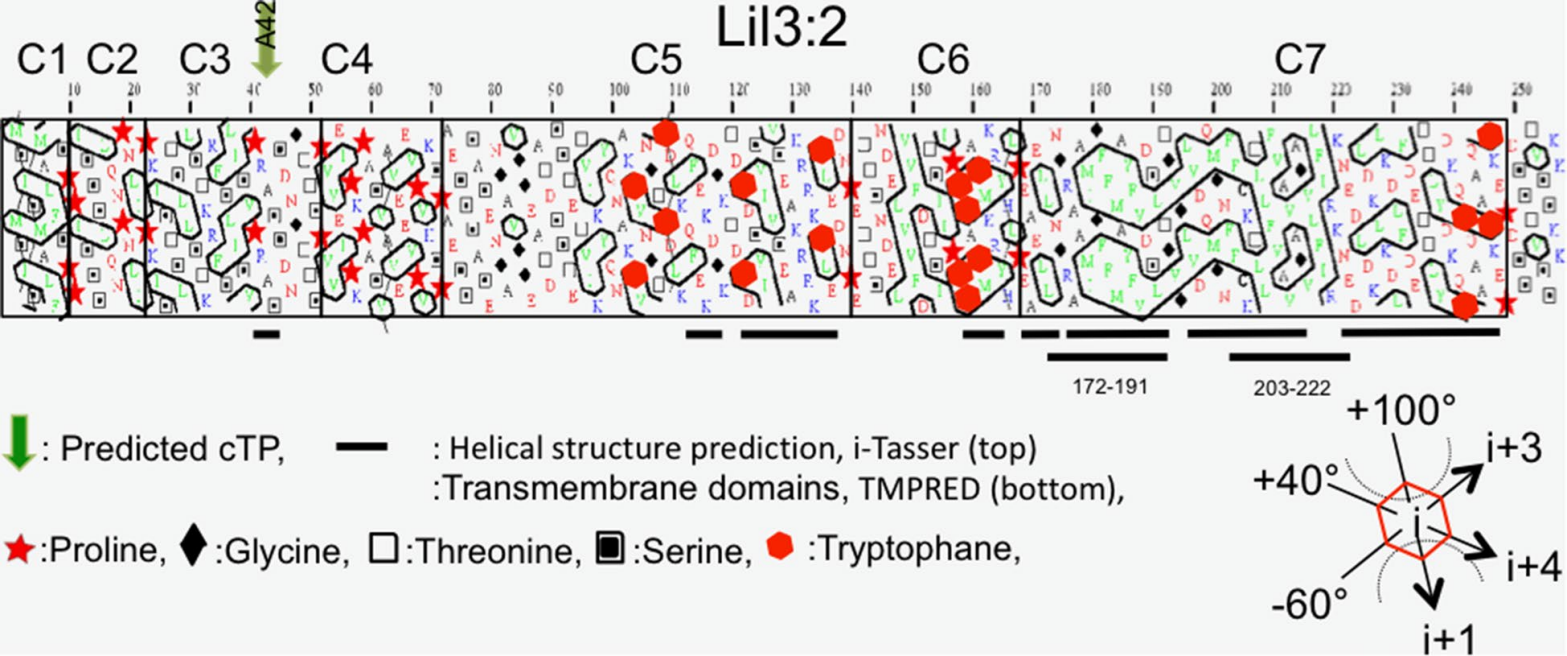
Hydrophobic cluster analysis and Trp-distribution in Lil3.2. The α-helical net duplicate of the amino acid sequences of LIL3:2 was analyzed and presented as hydrophobic cluster analysis (HCA) [25, 26]. Amino acids are plotted in one letter code and are colored or replaced by symbols [polar AA’s (N, D, E, Q), red; AA’s charged positively at neutral pH (K, R), blue; hydrophobic AA’s (F, I, V, L, W, M, Y), green]. Amino acids replaced by symbols are P, red star; T, empty square; S, square with smaller black square; G, black diamond, and W, filled red hexagon. Black outline highlights hydrophobic clusters and black lines under the plot were predicted as transmembrane regions by MPEx http://blanco.biomol.uci.edu/mpex/ (bottom) or as helical domains by i-Tasser (https://zhanglab.ccmb.med.umich.edu/I-TASSER/) (top). The HCA plot was subdivided according to the pattern of Prolin distribution in clusters (C) C1–C7. Key angular positions of amino acids relative to any amino acid (i) in an alpha-helical arrangement are provided in the direction of the protein sequence at + 100° (arrow, i + 1), − 60° (arrow, i + 3), and + 40° (arrow, i + 4) in the HCA plot

The peak in the first derivative of the thermogram is characterized as inflection or mid-point in the unfolding transition. It reports the melting temperature (Tm) at which the relative amounts of folded and unfolded protein are the same in two-stage denaturation. At this temperature in kelvin, the free energy change for denaturation is zero and equals the ratio of the change in enthalpy and entropy during denaturation. In nanoDSF, this peak is brought about by the spectral changes associated with the differential exposition of protein specific Trp-residues towards an environment changing with temperature [20]. For analysis of LIL3 unfolding it is considered that the label-free DSF signal reports about the change in protein domain structure and protein environmental exposure alike. Regarding the protein domain architecture of LIL3, dynamic structural analysis of LIL3 by TmPred (https://embnet.vital-it.ch/software/TMPRED_form.html) predicts a cytosolic N-terminal loop region and a C-terminal transmembrane region with two transmembrane segments at aa 172–191 and 203–222. In contrast, structural analysis using i-tasser (https://zhanglab.ccmb.med.umich.edu/I-TASSER/) predict eight helical domains of different length and proposes a helical alignment with proteins containing four transmembrane helical domains as widespread as the human nuclear pore complex (5IJO [21]) or the photoprotective protein PsbS (4RI2 [22]). LIL3.2 contains 7 cluster regions for Pro (Fig. 6) and two of the Pro clusters are directly at the border of two transmembrane regions at aa 172–191 and aa 203–222 which are predicted by TmPred and which are in accordance with the analysis of the LIL3 structure using hydrophobic cluster analysis (HCA, http://mobyle.rpbs.univ-paris-diderot.fr/cgi-bin/portal.py?form=HCA#forms::HCA) and show a high degree of overlap with the helical structure prediction by i-Tasser (Fig. 6). Predictions differ in part with respect to a helical localization of Trp containing domains of LIL3. Trps are distributed in Pro clusters C5, C6 and C7, with 5 of the 9 Trp residues arranged directly upstream and downstream of the proteins predicted two transmembrane alpha-helical domains in Pro cluster C6 and C7 (Fig. 6). Transmembrane regions predicted by TmPred and HCA are therefore completely Trp free, while Trp localization overlaps with most of the helical segments predicted according to HCA and i-Tasser. A short loop domain of 11 amino acids between transmembrane helix one and two in LIL3 as predicted by TmPred indicates that two Trp containing regions containing 5 of the 9 Trp residues of LIL3 are arranged directly next to the start and the stop of the two transmembrane alpha helix sequence (Fig. 6). In this configuration, all Trps of LIL3 are arranged on the same side of the membrane in the lipid headgroup region and could provide an effective floating means at the non-polar/polar interface on one side of the membrane [23] (Fig. 6). This indicated that the transmembrane regions of LIL3 could act as a thermostable membrane anchor that influences the stability of the polar loop regions [24]. The endogenous Trp fluorescence of LIL3 showed only one characteristic fast rise at a transition temperature with peak at 60 °C in the absence of oxidized DTT (Fig. 2). This indicated that Trp residues were exposed in one coherent thermal unfolding step in which unfolding of the alpha helical transmembrane domain was linked to a change in Trp exposure to the water phase (Fig. 2). However, in the presence of oxidized DTT, an interaction of DTT with the polar loop regions of LIL3 could be responsible for the observed step-wise temperature dependent exposure of the Trp residues. This would imply that unfolding of the transmembrane segment by heat became invisible because Trp residues were already unfolded at lower temperature based on the chemical interference with oxidized DTT. This would corroborate a localization of the transmembrane structures as predicted by TmPred and HCA and of helical regions associated to the transmembrane segments as predicted by i-Tasser (Fig. 2). We therefore conclude that the transmembrane helical domain in LIL3 maintains Trp containing domains folded toward the transmembrane domains of the LIL3 protein.

### The folding state of helical domains correlates with reconstitution of Chl *a*

**Fig. 7:**
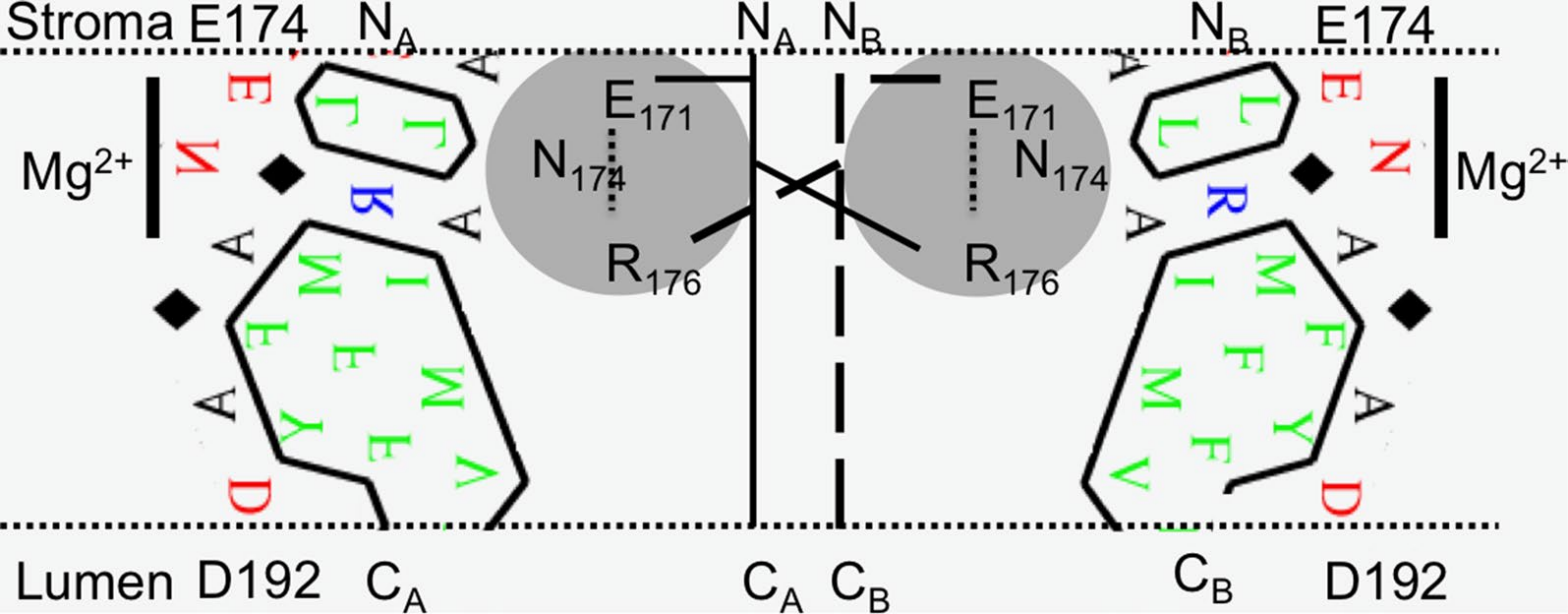
Model for Chl *a* binding to N174 and LIL3 dimerization. The predicted transmembrane helix 1 of LIL3 is displayed as HCA plot between amino-acid E174 to D192. The dimeric state is depicted by two helices plotted as mirror images to span the thylakoid membrane from the stroma to the lumen in the direction from N- to C-terminus (N_A_ to C_A_ and N_B_ to C_B_). The amino-acid Asn174 (N) binding Chl (black line, Mg^2+^) is pointing outward towards the membrane lipid phase. The graphical sketch in the center explains the contribution of amino-acids e E171 and R176 for interaction of LIL3 monomers by overlapping E–R ion-pairing (dashed line) and the proposed interaction for overlapping monomer interaction by N174 (helixA) and R176 (helixB) and N174 (helixB) and R176 (helixA) to explain the lack of LIL3 dimerization and Chl interaction in the LIL3 mutants

The heating requirement for successful reconstitution implies that the recombinant protein needs to be unfolded to establish an interaction with Chl. Also, all protocols for reconstitution of Chl binding to a membrane protein are conducted in the presence of a detergent with the implicit understanding that the detergent micelle stabilizes folding of the membrane protein domains and mimics the non-polar membrane phase [9]. Here, solubilization of LIL3 in DDM micelles has been investigated. Micelles were identified at about 10 nm in the reconstitution buffer (Fig. 2, Additional file 3: Fig. S2). The explicit decrease of the DDM intensity in the presence of LIL3, as well as of Chl *a* and also during reconstitution indicate that reconstitution components interact upon heating and successive RT incubation. This suggests that LIL3 undergoes a transition from a soluble state in the absence of Chl binding that is mainly determined by interaction with DTT towards a soluble state upon binding to Chl *a* that is accompanied with an interaction with DDM micelles (Fig. 1, Additional file 2: Fig. S1, Additional file 3: Fig. S2, Additional file 4: Fig. S3, Additional file 5: Fig. S4). Here, the overlapping distribution profile of the micellar signals and the differential increase in the range of particles with a h_d_ of about 2–5 nm and 160 nm were determined (Fig. 2b). The potential for interaction of LIL3 with Chl *a* is established by the differential effect of oxidized DTT on the solubility (Fig. 3b) and maintenance of LIL3 in a partly unfolded state at RT (Fig. 2). This could enable Chl *a* that is strictly found to dissolve in DDM micelles to access the Chl binding site. According to this understanding of the reconstitution process, oxidized DTT maintains the flexibility of the helical regions containing the Trp residues especially on one side of the transmembrane helices of LIL3. This coincides with the site at which the LHC motif is localized and N174 is exposed to the detergent phase (Figs. 6, 7). The selective influence of oxidized DTT is based on the structural changes associated with the change of DTT from the protonated state of a linear DTT molecule to its oxidized and cyclic six atom ring structure at pH 11. This structural change could make a direct interaction with the helical structures of the LIL3 protein possible and/or increase the proteins structural flexibility during protein folding by decreasing water entropy [27]. It could therefore be that the interactions of oxidized cyclic DTT with the protein and its surrounding water molecules provides the means for the stepwise increase in the affinity recorded during the 2-h incubation kinetics of a LIL3 reconstitution [9].

### Amino acid N174 is binding Chl in LIL3

The LIL3 mutant analysis showed that selected amino acids of the LHC motif responsible for Chl binding in LHC [5–8], also in part apply to Chl binding in LIL3. In the LIL3 N174A substitution mutant, Chl *a* binding was abolished, showing that N174 localized in the LHC motif consensus sequence is essential for Chl *a* binding to LIL3 (Fig. 5). The LIL3 E171A and R176A substitution mutants retained the ability to interact with Chl *a*, but with a six- and five-fold lower affinity (Fig. 4). Despite the determination of Kd for Chl binding in both mutants, thermophoretic mobility could not be saturated with LIL3 protein to reach a plateau. This suggests that also for the N174A substitution mutant a binding constant could have been determined if the recombinant mutants could be expressed and purified at higher concentration. Nevertheless, the LIL3 binding constant for Chl *a* is in general significantly weaker than for other photosystem Chl *a*-binding proteins indicating a different functional role of Chl binding to LIL3 [9, 10].

Chl molecules in LHC that are comparable to the evolutionary conserved binding site in LIL3 are bound to amino acids from two LHC motives located distant in primary sequence [17, 28]. For LIL3, dimerization has previously been suggested to establish the binding site despite the absence of a second LHC motif and Chl *a* binding to LIL3.2 had been suggested to precede dimerization in WT LIL3 in vitro [10]. The significant reduction in Chl *a* binding strength for recombinant LIL3 E171A, and R176A mutants could therefore be explained by the finding that both mutant proteins were no longer able to dimerize (Additional file 7: Fig. S6). Interestingly, substitution mutant N174A lacked both dimerization and Chl binding. This implied that besides amino acids R176 and E171 also N174 is required to establish the dimerization site. We therefore propose an extension of our previously suggested model [10] to underline the importance of all three amino acids of the LHC binding motif for dimerization and to establish Chl binding to N174 in LIL3 (Fig. 7).

### LIL3 may have a structural, but different functional evolution compared to LHCP

The LHC motif is conserved from the early one-helix cyanobacterial HLIPs via the two-helical SEPs to the three-helical LHCs [1]. It has been discussed that the one-helical cyanobacterial HliD forms a dimer and in its dimeric form binds six chlorophylls and two β-carotenes [29]. We have proposed that hetero-dimerization of the two-helical LIL3 protein precedes binding of two molecules of Chl *a* and that Chl *a* binding is independent of the presence of carotenoids [9, 10]. Our current data strongly support the previous findings that in LIL3, dimerization establishes a foundation for effective Chl binding as shown here by residue N174 which is predicted to be positioned on opposite faces of the transmembrane domains in LIL3; while, in LHCP dimerization of two LHC motives facilitate Chl binding (Fig. 7).

LHC has been proposed to descend from an internal gene duplication of the SEPs [1]. According to present understanding, the pool of two-helix SEPs diverged into a diverse protein group consisting of early light induced proteins (ELIPs), PSBS and LHC in plantae [1]. The indications of an inherent need for dimerization of LIL3 and HliD to facilitate Chl binding, could explain the advantage of genetically diverged proteins ELIPs, PSBS and LHCs. In LHC2, the proposed internal gene duplication of transmembrane helices 1 and 3 resulted in the duplication of the LHC motif and formation of a structural basis for increased chlorophyll binding capacity (14/monomer). If positioned in evolutionary context, interaction of the amino-acids in the internal LHC motif can be regarded as a hetero-dimerization. Together with the hetero-trimerization of LHC isomers in the LHC-trimer the Chl binding capacity of LHC increases to 42 molecules, with 24 Chl *a* and 18 Chl *b* per complex [17]. However, if scaled down to the Chl binding LHC motif as characterized in LIL3, also in LHC only one Chl molecule is directly bound per motif [9]. For LIL3, the predicted dimerization of the two transmembrane a-helices via the Chl binding amino acids of the LHC motif indicates a structural conservation of the motif but different evolution of the proteins function.

## Conclusions

A combination of several different methods have been applied to characterize Chl interaction with LIL3. The combination of results from MST and native PAGE both support that amino acid N174 is binding Chl *a* in LIL3. The combination of results from DLS and nanoDSF clarified the change in the interaction of LIL3 and Chl *a* with DTT and DDM and of Chl *a* with DDM micelles and the transition during the reconstitution steps have been followed. The strategy significantly increased our functional understanding of the Chl protein interaction and how to characterize in vitro reconstitution further.

## Materials and methods

### Aim and design of study

This study was directed to improve our understanding of the principles and mechanisms of reconstitution, a frequently used method in plant biology for analysis of protein-pigment interaction. We have previously contributed to modify the reconstitution protocol [9, 10] and established MST, DLS and ELS in our laboratory to elucidate the basis of the molecular interactions. NanoDSF experiments were performed by AMJ at NanoTemper Technologies GmbH (Munich, Germany).

### Protein expression and purification

Site directed LIL3.2 (AT5G47110) mutants (LIL3.2 E171A, N174A and R176A) were purchased from life technologies in a pMA-T vector, were further amplified by forward and revers primers gtcatatgatgtctatatccatggcgt and cctaggtcacttctttgaagaaac respectively and transferred to the pET151d vector (Invitrogen) by Topo cloning. Mutants were expressed with a N-terminal His-tag in *E. coli* BL21 [F–ompT hsdS(rB– mB–) gal dcm λ(DE3)] and harvested as described in [10]. LIL3 mutants were purified as in [9]. The purified proteins were separated by SDS PAGE [12% (w/v)], stained with Coomassie Brilliant Blue (CBB) and blotted with a His (1:3000, Sigma Aldrich) primary antibody as described in [10].

### Reconstitution assays

Reconstitution assays were performed based on protocols as described [4, 5, 9]. For both LIL3.1 and LIL3.2 isoforms from Arabidopsis thaliana, very similar results were obtained [10]. Based on the higher purification yield only experimental work with LIL3.2 is shown here. In brief; LIL3 inclusion bodies (30 μM) were solubilized in a reaction buffer containing 100 mM Tris pH 11, 5 mM 6-aminocaproic acid, 1 mM benzamidine and 12.5% sucrose and *n*-Dodecyl β-d-maltoside (DDM) at a final concentration of 6 mM DDM respectively. 100 mM DTT and 6 μM Chl *a* (solubilized in diethyl ether/Ethanol 1:1) were added prior to heating samples to 100 °C for 1 min followed by a 2-h incubation at RT in the dark.

### Dynamic light scattering, DLS

Dynamic light scattering (DLS) was applied to determine if LIL3 was integrated to the DDM micelle structure during reconstitution using a Zetasizer Nano ZSP (Malvern, UK). The intensity distribution profile [diameter (d. nm)] of Chl *a* and Chl *a* reconstituted with LIL3.2 was recorded upon dissolving reaction components in reconstitution buffer containing 6 mM DDM and 100 mM DTT at 25 °C. Recording was conducted when all reaction components were assembled, 1 min after boiling, and 2 h after boiling under reconstitution conditions (100 mM Tris, 5 mM 6-aminocaproic acid, 1 mM benzamidine and 12.5% sucrose, 100 mM DTT, pH 11). The average of the peak value weighted by the mean hydrodynamic diameter is given for all samples. In dynamic light scattering (DLS) analysis, the mean hydrodynamic diameter of particles (h_d_) can be determined from the particles characteristic Brownian motion. An intensity distribution of determined h_d_-values displays the dispersity of the sample. Care has to be taken to interpret concentration changes of sample as changes of the intensity values are proportional to h_d_^6^ [30]. Cumulative analysis is not valid for poly-disperse samples. Therefore, the distribution means in the linear range is reported, not Z-Average and PDI [31–35].

### Thermal unfolding, nanoDSF

Samples for thermal unfolding were prepared by combining the same volume of LIL3.2 sample (5 μM) in DDM micelles (6 mM) in reconstitution buffer (100 mM Tris, 5 mM 6-aminocaproic acid, 1 mM benzamidine and 12.5% sucrose, pH 11) in the absence or presence of DTT. Fluorescence based thermal experiments were performed using Prometheus NT.48 (NanoTemper Technologies, Germany). All capillaries containing 10 μl LIL3.2 recombinant protein were sealed. The temperature was increased by a rate of 1 °C/min from 20 to 110 °C and the fluorescence at emission wavelengths of 330 nm and 350 nm was measured. For interpretation of spectra, application notes from the company were considered.

### Electrophoretic light scattering, ELS

Electrophoretic light scattering was applied to determine the zeta potential (stability) of the DDM micelle, LIL3 dimer, Chl *a* and LIL3 reconstituted with Chl *a* following the reconstitution procedure. Experiments were performed in triplicate and the average plotted. Standard deviation was calculated for the three independent measurements.

### Microscale thermophoresis, MST

The intrinsic fluorescence of Chl *a*, was monitored at a final concentration of 30 nM Chl *a* diluted in reconstitution buffer containing 6 mM DDM, while non-fluorescent LIL3.2 mutants E171A, N174A and R176A were titrated in a 1:1 dilution series (concentrations between 2.500 µM and 0.61 nM) in reconstitution buffer containing 6 mM DDM. After a 2-h incubation at RT, samples were loaded into Monolith™ NT.115 MST Premium Coated Capillaries (NanoTemper Technologies, Munich, Germany) and measured using a Monolith NT.115 at RT and analyzed by the MO. Control Software, LED/excitation 10%, MST power setting 40%. Results are recorded as normalized fluorescence (F_norm_ = F_hot_/F_initial_) and presented as differential fluorescence [ΔF_norm_ = F_norm_ (bound) − F_norm_ (unbound)] which reads as ΔF_norm_ = baseline corrected F_norm_ (‰) [36]. Lil3.2 mutant dimer analysis was performed as described for wild type LIL3.2 [10].

### Native PAGE

LIL3.2 mutants bound to Chl *a* was isolated by LDS-Native (LN) PAGE on 3–12% polyacrylamide gels (Novex, Life technologies, California USA) with a cathode buffer supplemented with 74 μM LDS [2]. Pigment and pigment binding protein were detected before protein staining by fluorescence scanning at 800 nm in a LI-COR Odyssey^®^ CLx and stained by CBB as described [10].

### Absorbance spectroscopy

Absorbance spectra were recorded from 200 to 400 nm (Shimadzu UV–VIS 2401PC, Duisburg, Germany). Spectra of DTT were measured at pH 6 in dH_2_O and at pH11 in reconstitution buffer. Spectra of LIL3.2 were recorded upon solubilization in 6 mM DDM, pH 11 in reconstitution buffer and in the presence or absence of DTT and upon reconstitution with Chl *a*.

## Additional files

**Additional file 1: Table S1.** The diameter of particles (values in nm) was recorded from samples in reconstitution buffer (methods) in the presence of DDM and Chl a (DDM CHLA), DTT and LIL3 (DTT LIL3), DDM, or DTT. Sample diameters were recorded after assembly of the reaction components (0H), 1 min after heating (100 °C) (0H B), and two hours after heating (2H B). The diameters from the mulitmodal polydisperse mean peak intensity profiles of each sample were arranged according to the intensity values as 1 (highest), 2 (middle), 3 (lowest) intensity (Peak).

**Additional file 2: Fig. S1.** Size determination of DDM and DTT. The intensity distribution profile (diameter (d. nm)) of 6 mM DDM (A) and of 100 mM DTT (B) at 0 time point (0H), at 0 time point after boiling (0H B) and 2 h after boiling (2H B) in reconstitution buffer (100 mM Tris, 5 mM 6-aminocaproic acid, 1 mM benzamidine and 12,5% sucrose, pH 11).

**Additional file 3: Fig S2.** Size determination of Chl a and LIL3. The intensity distribution profile (diameter (d. nm)) of Chl a solubilized in 6 mM DDM (A) and LIL3 solubilized in DTT at 0 time point (0H), at 0 time point after boiling (0H B) and 2 h after boiling (2H B) in reconstitution buffer (100 mM Tris, 5 mM 6-aminocaproic acid, 1 mM benzamidine and 12.5% sucrose, pH 11).

**Additional file 4: Fig S3.** Size determination of DDM and DTT with and without LIL3. The intensity distribution profile (diameter (d. nm)) of 100 mM DTT and 6 mM DDM (A) and of LIL3 solubilized in 100 mM DTT and 6 mM DDM (B) at 0 time point (0H), at 0 time point after boiling (0H B) and 2 h after boiling (2H B) in reconstitution buffer (100 mM Tris, 5 mM 6-aminocaproic acid, 1 mM benzamidine and 12.5% sucrose, pH 11).

**Additional file 5: Fig S4.** LIL3 is co-localized with DDM micelles when reconstituted with Chl a. The intensity distribution profile (diameter (d. nm)) of Chl a (A) and Chl a reconstituted with LIL3.2 (B) solubilized in 6 mM DDM micelles with 100 mM DTT at 0 time point (0H) at 0 time point after boiling (0H B) and 2 h after boiling (2H B) under reconstitution conditions (100 mM Tris, 5 mM 6-aminocaproic acid, 1 mM benzamidine and 12.5% sucrose, 100 mM DTT, pH 11).

**Additional file 6: Fig S5.** DTT induces a stepwise thermal unfolding of LIL3 at pH 11. The first derivative was calculated from the fluorescence ratio changes (Δ(*F*350 nm/*F*330 nm)/Δ*T*) determined as a function of temperature (°C), for LIL3 solubilized by DDM in reconstitution buffer in the absence and presence of DTT at pH 11 (A, pH 11 and pH 11, DTT) and pH 6 (B, pH 6 and pH 6, DTT), respectively.

**Additional file 7: Fig S6.** LIL3 mutants do not dimerize with WT LIL3. Lil3 mutants E171A, N174A and R176A were solubilized in DDM micelles (6 mM) at increasing concentrations 0.61 nM–5 µM in the presence of a constant concentration (1 µM) of fluorescently labelled WT LIL3.2-NT647. Normalized fluorescence difference from three MST measurements were plotted against the LIL3.2 E171A (A), LIL3.2 N174A (B) and LIL3.2 R176A (C) concentrations.
