# Supplementary figures and images for: A strategy to characterize chlorophyll protein interaction in LIL3
Source: Plant Methods. 2019 Jan 5;15:1. doi: 10.1186/s13007-018-0385-5 (PMC6320596; doi:10.1186/s13007-018-0385-5)

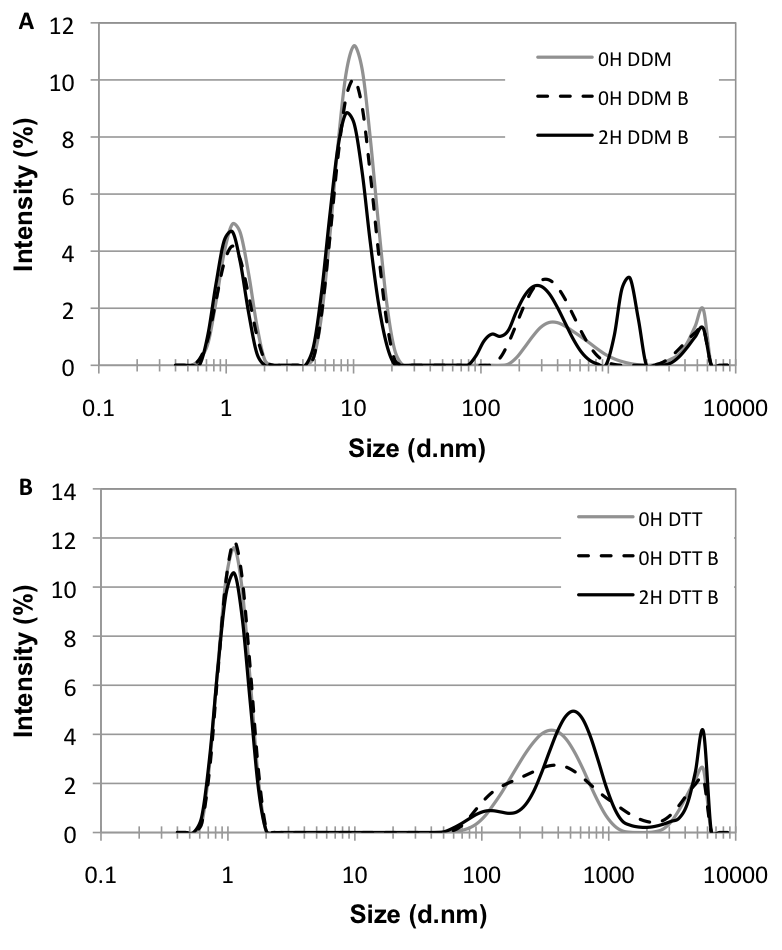

Supplement: Supplementary file 2 — Additional file 2: Fig. S1. Size determination of DDM and DTT. The intensity distribution profile (diameter (d. nm)) of 6 mM DDM (A) and of 100 mM DTT (B) at 0 time point (0H), at 0 time point after boiling (0H B) and 2 h after boiling (2H B) in reconstitution buffer (100 mM Tris, 5 mM 6-aminocaproic acid, 1 mM benzamidine and 12,5% sucrose, pH 11). [file 13007_2018_385_MOESM2_ESM.tiff]

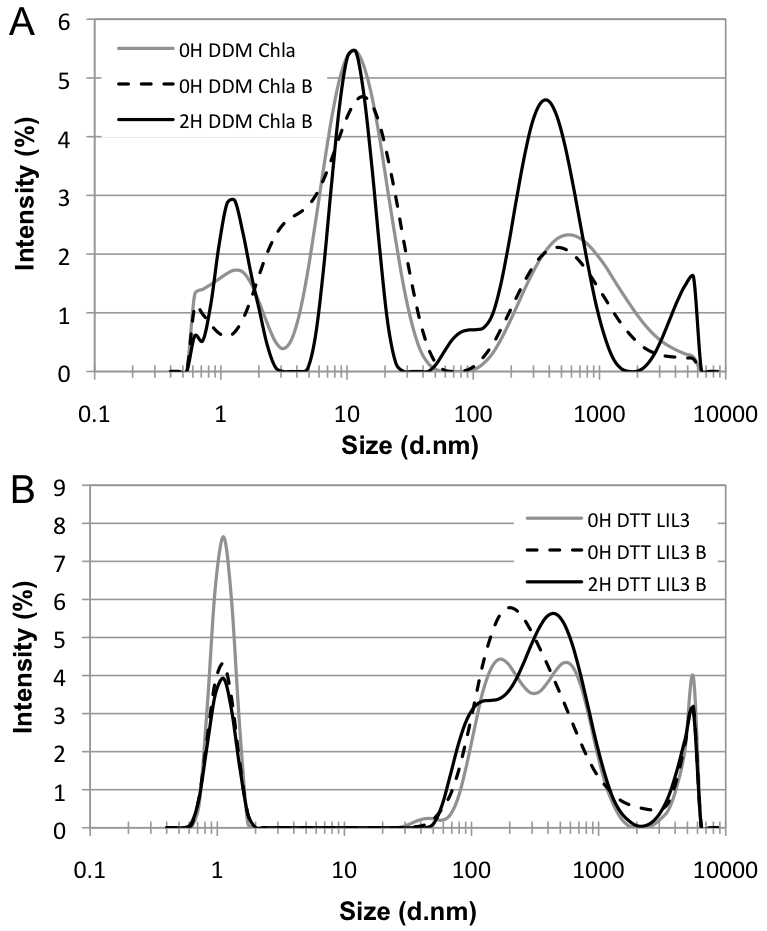

Supplement: Supplementary file 3 — Additional file 3: Fig S2. Size determination of Chl a and LIL3. The intensity distribution profile (diameter (d. nm)) of Chl a solubilized in 6 mM DDM (A) and LIL3 solubilized in DTT at 0 time point (0H), at 0 time point after boiling (0H B) and 2 h after boiling (2H B) in reconstitution buffer (100 mM Tris, 5 mM 6-aminocaproic acid, 1 mM benzamidine and 12.5% sucrose, pH 11). [file 13007_2018_385_MOESM3_ESM.tiff]

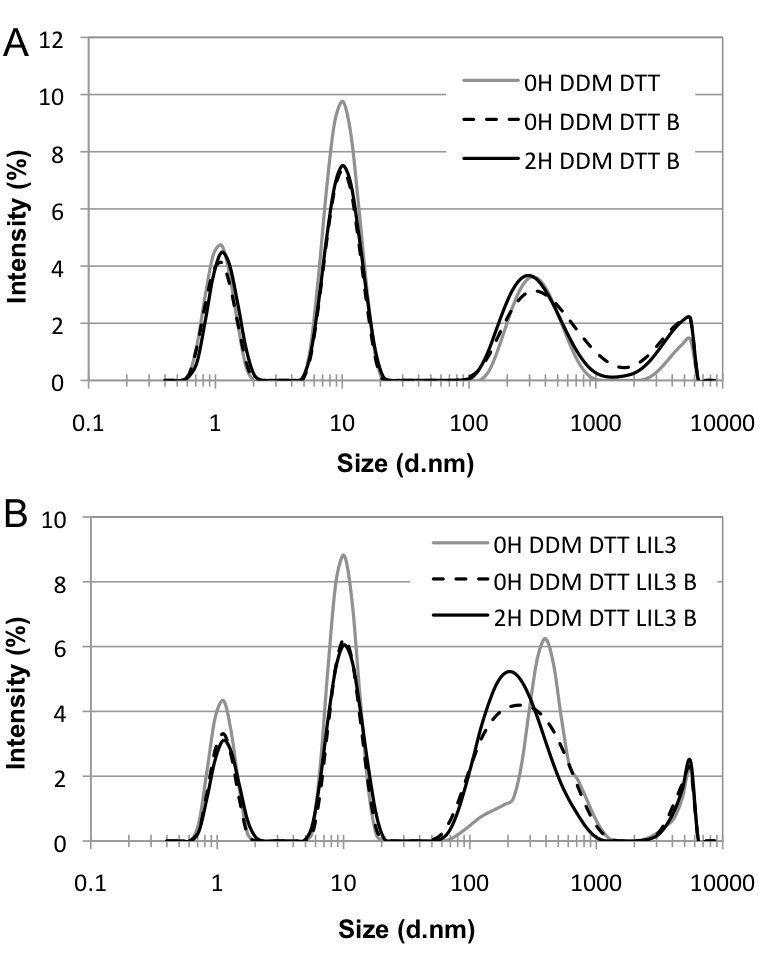

Supplement: Supplementary file 4 — Additional file 4: Fig S3. Size determination of DDM and DTT with and without LIL3. The intensity distribution profile (diameter (d. nm)) of 100 mM DTT and 6 mM DDM (A) and of LIL3 solubilized in 100 mM DTT and 6 mM DDM (B) at 0 time point (0H), at 0 time point after boiling (0H B) and 2 h after boiling (2H B) in reconstitution buffer (100 mM Tris, 5 mM 6-aminocaproic acid, 1 mM benzamidine and 12.5% sucrose, pH 11). [file 13007_2018_385_MOESM4_ESM.tiff]

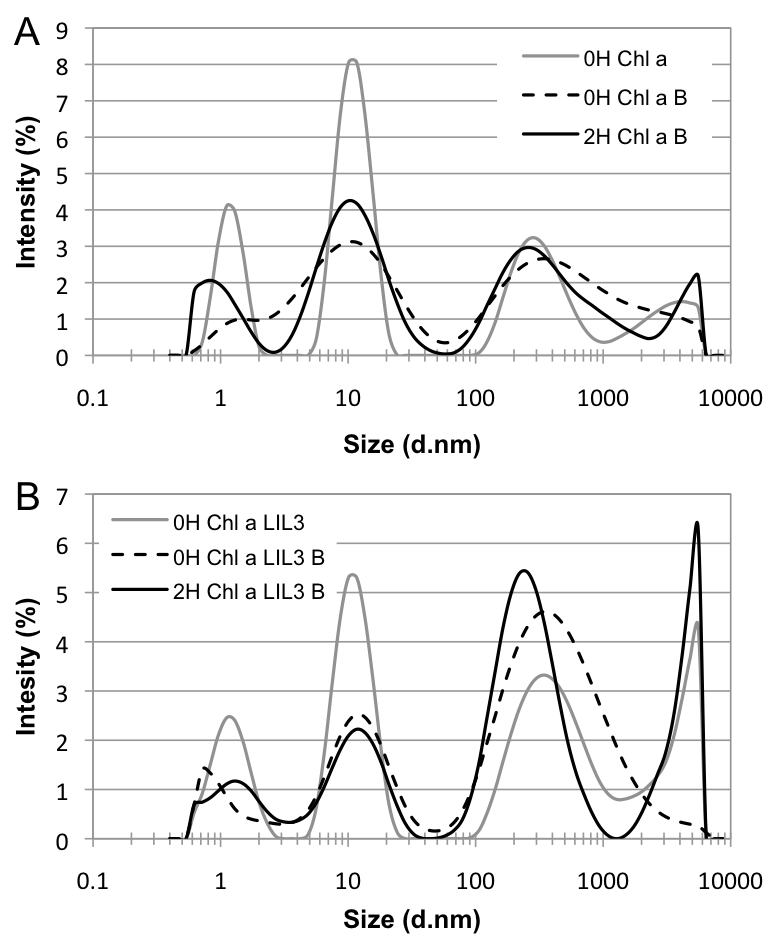

Supplement: Supplementary file 5 — Additional file 5: Fig S4. LIL3 is co-localized with DDM micelles when reconstituted with Chl a. The intensity distribution profile (diameter (d. nm)) of Chl a (A) and Chl a reconstituted with LIL3.2 (B) solubilized in 6 mM DDM micelles with 100 mM DTT at 0 time point (0H) at 0 time point after boiling (0H B) and 2 h after boiling (2H B) under reconstitution conditions (100 mM Tris, 5 mM 6-aminocaproic acid, 1 mM benzamidine and 12.5% sucrose, 100 mM DTT, pH 11). [file 13007_2018_385_MOESM5_ESM.tiff]

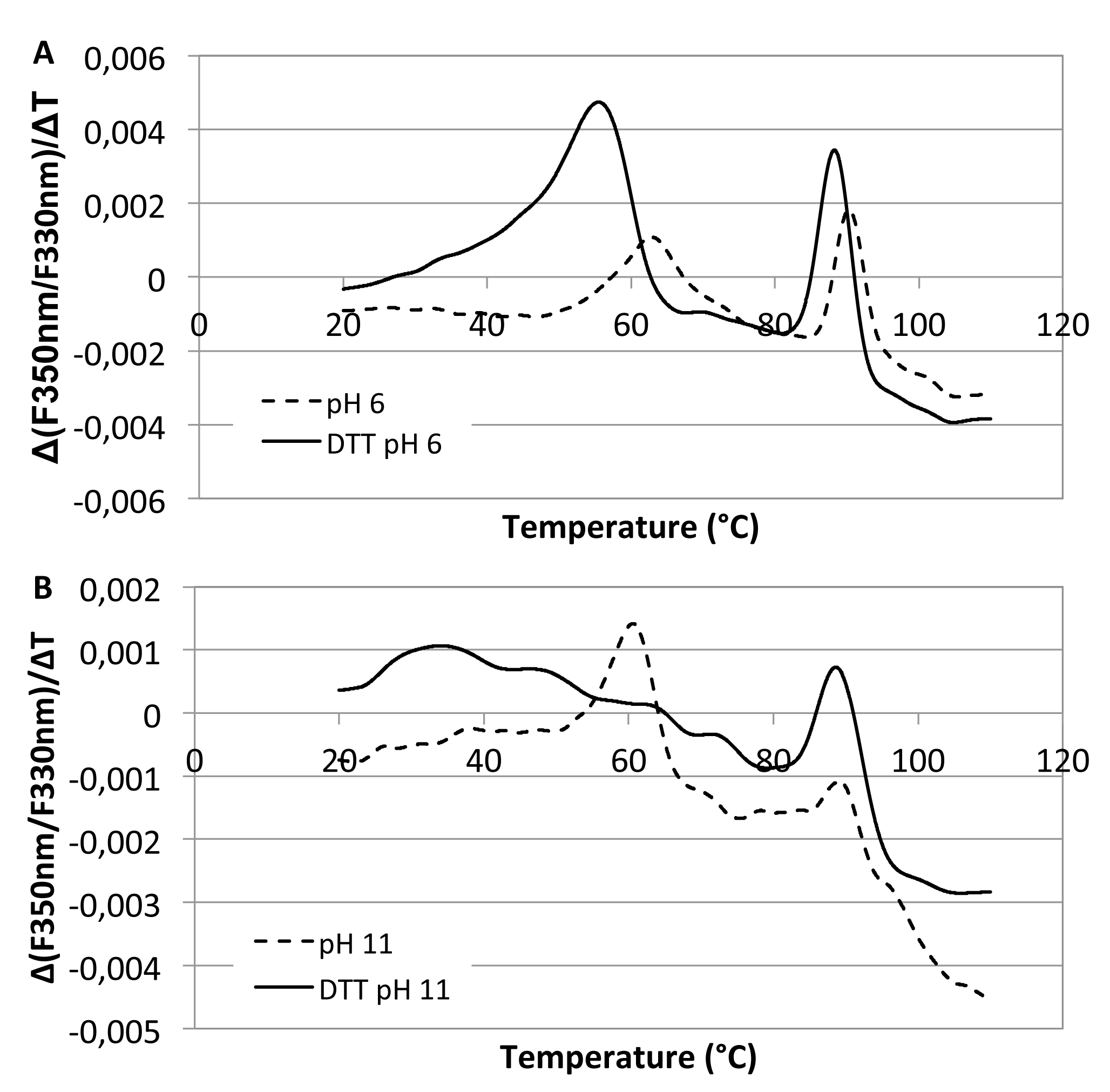

Supplement: Supplementary file 6 — Additional file 6: Fig S5. DTT induces a stepwise thermal unfolding of LIL3 at pH 11. The first derivative was calculated from the fluorescence ratio changes (Δ(F350 nm/F330 nm)/ΔT) determined as a function of temperature (°C), for LIL3 solubilized by DDM in reconstitution buffer in the absence and presence of DTT at pH 11 (A, pH 11 and pH 11, DTT) and pH 6 (B, pH 6 and pH 6, DTT), respectively. [file 13007_2018_385_MOESM6_ESM.tif]

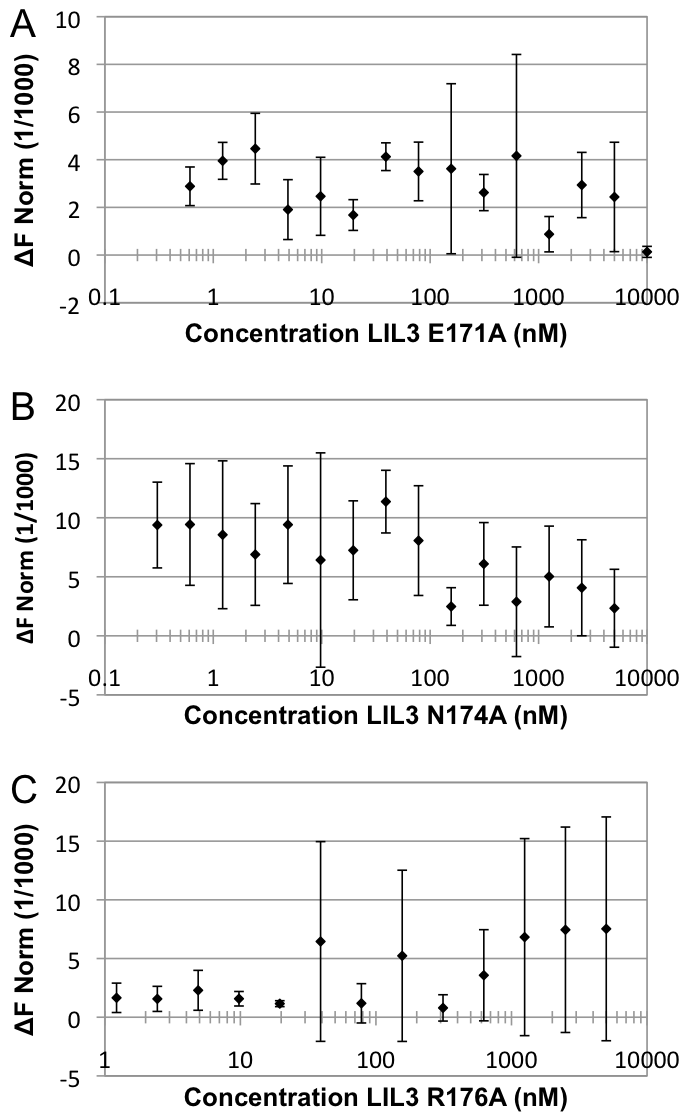

Supplement: Supplementary file 7 — Additional file 7: Fig S6. LIL3 mutants do not dimerize with WT LIL3. Lil3 mutants E171A, N174A and R176A were solubilized in DDM micelles (6 mM) at increasing concentrations 0.61 nM–5 µM in the presence of a constant concentration (1 µM) of fluorescently labelled WT LIL3.2-NT647. Normalized fluorescence difference from three MST measurements were plotted against the LIL3.2 E171A (A), LIL3.2 N174A (B) and LIL3.2 R176A (C) concentrations. [file 13007_2018_385_MOESM7_ESM.tiff]
